# Unilateral papilledema and peripapillary polypoidal choroidal vasculopathy as the presenting manifestations of intracranial hypertension

**DOI:** 10.5935/0004-2749.20210098

**Published:** 2025-08-21

**Authors:** Aline Mota Freitas Matos, Leonardo Provetti Cunha, Ana Cláudia F. Suzuki, Luiz Guilherme Marchesi Mello, Rony C. Preti, Leandro C. Zacharias, Mário L. R. Monteiro

**Affiliations:** 1 Division of Ophthalmology, Faculdade de Medicina, Universidade de São Paulo, São Paulo, SP, Brazil; 2 Faculdade de Medicina, Universidade Federal de Juiz de Fora, Juiz de Fora, MG, Brazil

**Keywords:** Meningioma, Choroidal neovascularization, Papilledema, Pseudotumor cerebri, Vision disorder, Meningioma, Neovascularização de coroide, Papiledema, Pseudotumor cerebral, Transtorno da visão

## Abstract

We have reported here the case of a 54-year-old woman with intracranial hypertension that presented with the unique features of unilateral papilledema and peripapillary polypoidal choroidal vasculopathy. Our investigations lead to the diagnosis of idiopathic intracranial hypertension and an incidental small right frontal meningioma. The patient was accordingly treated with oral acetazolamide, followed by three consecutive monthly intravitreal injections of bevacizumab, which resulted in the inactivation of the polypoidal choroidal vasculopathy, marked reduction of lipid exudation, and complete absorption of the subretinal fluid. This case serves as the first documentation of polypoidal choroidal vasculopathy associated with papilledema. It also demonstrates that choroidal vascular abnormalities may occur even when optic disk edema is unilateral, which is an uncommon manifestation of increased intracranial pressure. Prompt recognition of such findings and its appropriate management are essential for adequate treatment and prevention of irreversible visual loss.

## Abstract (pt)

Relatamos um caso de uma paciente de 54 anos com hipertensão intracraniana que apresentava achados atípicos de papiledema unilateral e vasculopatia polipoidal da coroide peripapilar. A investigação levou ao diagnóstico de hipertensão intracraniana idiopática e de um pequeno meningioma incidental. A paciente foi tratada com acetazolamida por via oral, seguida de três injeções intravítreas mensais de bevacizumabe, resultando em inatividade da vasculopatia polipoidal da coroide, redução da exsudação e completa absorção do líquido subretiniano. A apresentação deste caso serve para documentar pela primeira vez vasculopatia polipoidal da coroide associada a papiledema. Ele também demonstra que podem ocorrer anormalidades vasculares da coroide mesmo quando o edema do disco óptico é unilateral, uma manifestação incomum do aumento da pressão intracraniana. O reconhecimento imediato desses achados e seu manejo adequado são essenciais para o tratamento adequado e para prevenção da perda visual irreversível.

## INTRODUCTION

Papilledema, the funduscopic sign of blurred and elevated optic disk margins, is indicative of increased intracranial pressure (ICP), possibly caused by ominous neurological diseases, including intracranial tumors, hemorrhages, infections, and the blockage of the cranial ventricular drainage system. When the symptoms of increased ICP, such as headache, are not prominent, differentiating papilledema from other optic neuropathies may be challenging; nevertheless, the fact remains that papilledema is generally bilateral and associated with preserved visual function^(1)^. However, uncommonly papilledema may be unilateral^(2)^, consideration of which causes diagnostic confusion. The diagnosis may also be difficult when papilledema is associated with visual loss, usually from progressive retinal nerve fibers loss leading to constricted visual fields (VF)^(3)^ and rarely from macular exudates and hemorrhages, choroidal folds, or neovascularization^(4)^, which usually occurs in chronic cases, most often in patients with idiopathic intracranial hypertension (IIH).

IIH is diagnosed based the following criteria: i) symptoms and signs of increased ICP papilledema; ii) elevated cerebrospinal fluid (CSF) opening pressure; iii) normal CSF analysis; iv) no imaging evidence of a structural cause for increased ICP; and v) no other cause of increased ICP^(5)^. This definition does not exclude the presence of unilateral optic disk swelling or concomitant unrelated intracranial tumors^(6)^.

The purpose of the present paper is to describe a patient that presented with a unique combination of unilateral papilledema and peripapillary polypoidal choroidal vasculopathy (PCV) in a patient with IIH and an incidental meningioma. By first reporting PCV associated with papilledema, our aim was to emphasize the need for prompt recognition and the treatment of such occurrence to prevent visual loss.

## CASE REPORT

A 54-year-old female obese patient was referred for evaluation of painless optic disk edema in the right eye (OD). She denied headache or permanent visual loss, but reported episodes of transient visual obscuration in the OD, which is usually triggered by postural changes. Past medical history also showed significant hypertension and depression.

**Figure 1:**
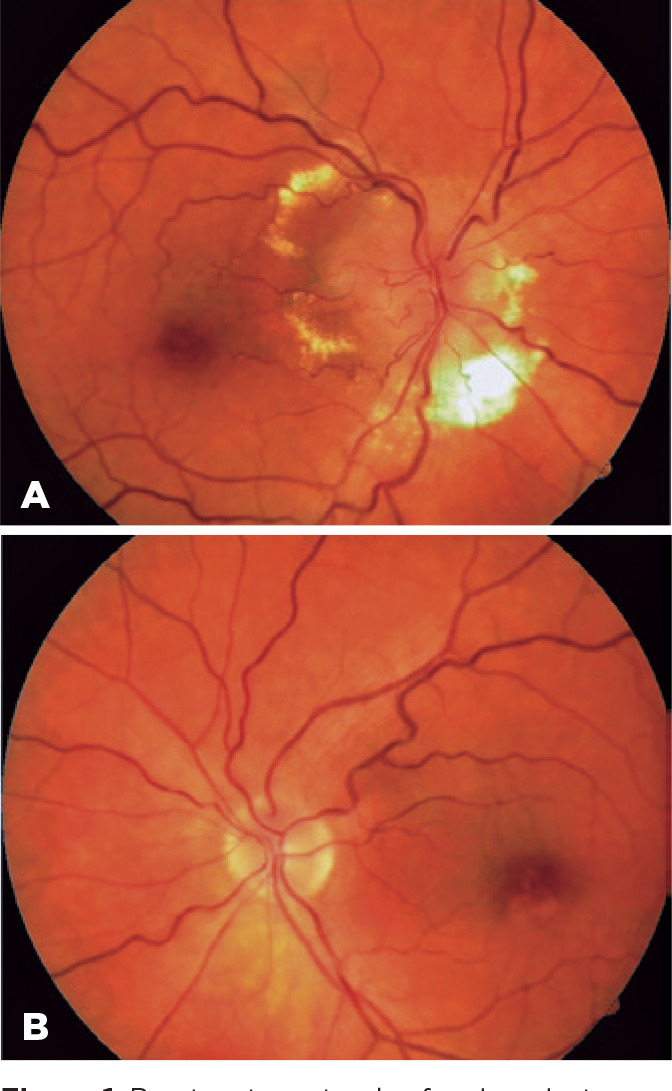
Pre-treatment color fundus photographs showing optic disc edema, exudates, and a reddish-orange peripapillary subretinal nod ular lesion in the right eye (A). Normal disc appearance and mild perifoveal pigmentary abnormality can be seen in the left eye (B).

**Figure 2:**
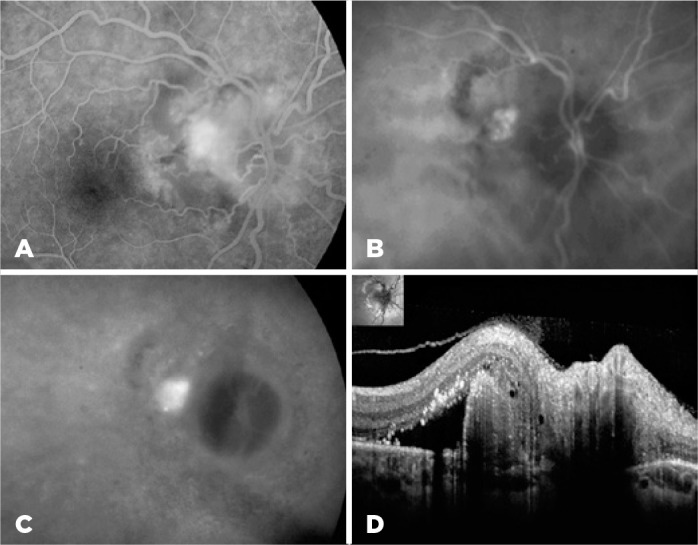
Multimodal imaging of the right eye. Fluorescein angiography (A) demonstrates a hyperfluorescent peripapillary nodule. Mid-phase indocyanine green angiography (B) reveals a cluster of peripapillary hyperfluorescent polyps surrounded by a dark hypofluorescent halo, blurring its contours in the late-phase to (C) indicate choroidal vascular hyperpermeability. OCT cross-sectional scan (D) along the peripapillary nodule can be seen as a thumb-like retinal pigment epithelium detachment, adjacent serous retinal detachment, and hard exudates.

On examination, best-corrected visual acuity (VA) was 20/20 in both eyes (OU), normal pupillary reactions, extraocular movements, biomicroscopy, and intraocular pressure. Ophthalmoscopy revealed optic disk edema surrounded by peripapillary exudates and a reddish-orange peripapillary subretinal nodular lesion temporally in OD and mild perifoveal retinal pigment epithelium (RPE) changes in the left eye (OS) (Figure 1). Fluorescein angiography demonstrated a peripapillary nodular hyperfluorescence with pronounced leakage of dye in OD (Figure 2A). Indocyanine green angiography (ICGA) of the OD showed a peripapillary PCV (Figure 2, B-C). The cross-sectional scan of optical coherence tomography (OCT) of the peripapillary nodule revealed a dome-shaped RPE detachment (PED) with moderate internal reflectivity and adjacent serous retinal detachment with multiple hyperreflective dots in the outer retina (intraretinal exudates) (Figure 2D). VF examination revealed an enlarged blind spot in OD. Examinations were unremarkable in OS.

**Figure 3:**
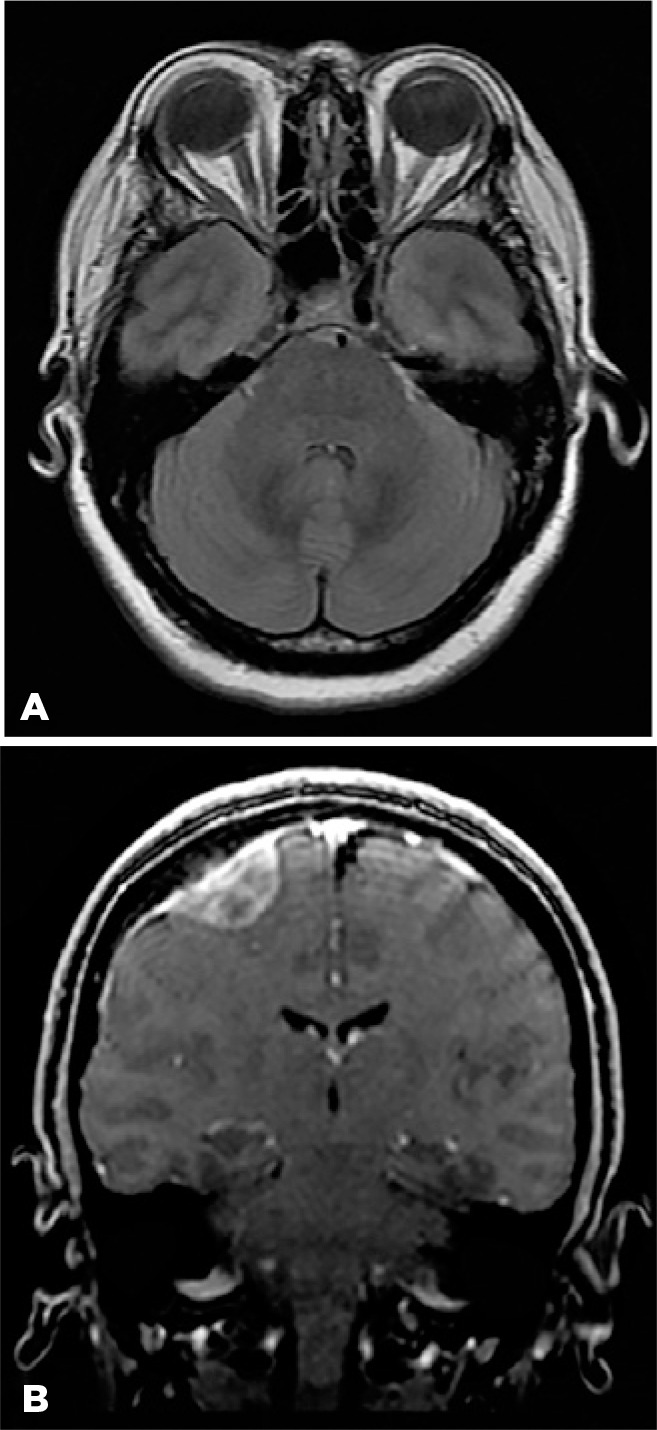
Cranial magnetic resonance imaging reveals flattening of the posterior pole of the right eye (A) and a small space-occupying lesion in the high right frontal lobe with a characteristic tail sign at its dural base compatible with a meningioma (B).

Neurological examination and extensive laboratory investigations were normal. Cranial magnetic resonance imaging (MRI) revealed flattening of the posterior pole of the OD and an empty sella, without ventricular enlargement or midline shift of the brain. There was a right frontal extra-axial mass with a tail sign at its dural base that was compatible with meningioma (Figure 3). There was no evidence of cerebral venous sinus thrombosis or stenosis. Lumbar puncture indicated an opening CSF pressure of 38 cmH_2_O with normal cytochemical analysis. A diagnosis of IIH with an incidental meningioma was accordingly made.

**Figure 4:**
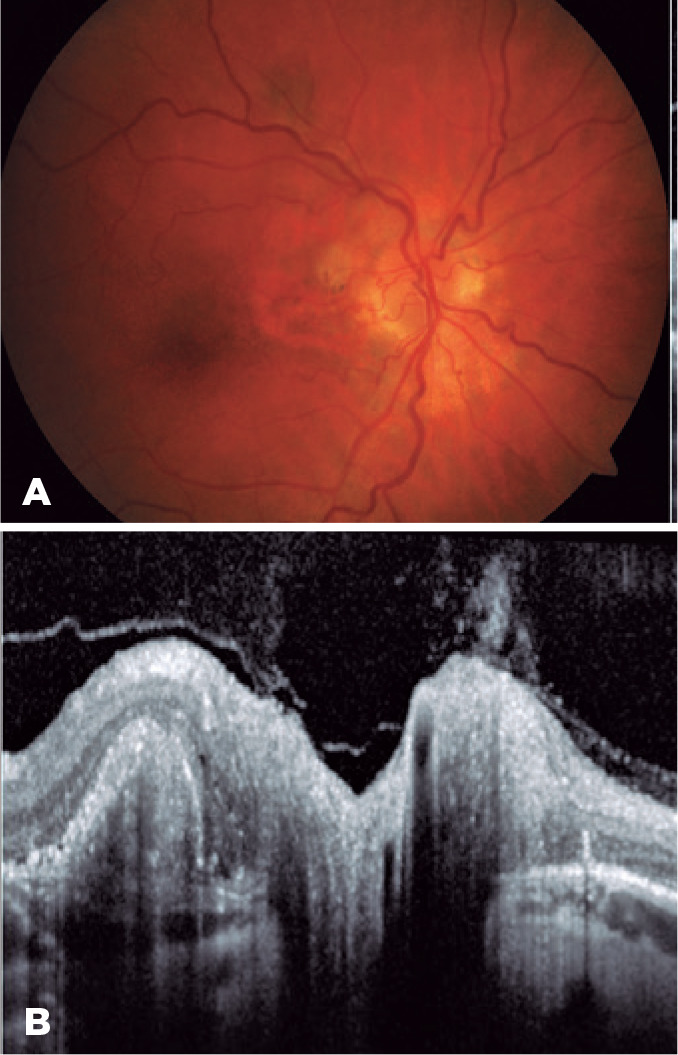
Color fundus photograph (A) and OCT (B) of the right eye after treatment. The nodular lesion became yellowish-white in color with adjacent retinal pigment epithelium atrophy, without optic disc edema, exudates, and subretinal fluid, although the thumb-like retinal pigment epithelium detachment persisted on the OCT line-scan (B).

Treatment with oral acetazolamide (750 mg/day) led to the disappearance of transient visual obscurations, but no improvement of the fundus findings were noted 2 months later. Subsequently, acetazolamide was maintained and three consecutive, monthly intravitreal injections of bevacizumab (1.25 mg/0.05 mL) were performed in OD, with complete absorption of the subretinal fluid, exudation, and optic disk edema (Figure 4). Weight reduction was gradually attained and acetazolamide tapered without recurrence of optic disk edema or PCV during a 7-year follow-up period. Repeat MRI scan demonstrated an unchanged meningioma, while CSF opening pressure reduced to 22 cmH_2_O.

## DISCUSSION

The present case presented a set of unusual associations that may confound the correct diagnosis of IIH. Although it is not unusual for papilledema to be asymmetric, the presentation of unilateral papilledema is uncommon,^(2)^ which may lead to confusion with other optic neuropathies such as optic neuritis or anterior ischemic optic neuropathy. Despite the unilateral presentation, the clinical findings and subsequent testing revealed findings that were indicative of IIH: an obese woman with transient visual obscuration, normal VA, enlargement of the blind spot, normal CSF analysis with elevated opening pressure, and cranial MRI documenting an empty sella and excluding other conditions that may explain an increased ICP. Although several mechanisms have been suggested to explain unilateral papilledema, such as congenital anomaly of the optic nerve sheaths, the loss of lamina cribrosa compliance or asymmetry of the optic canals^(7)^, the exact cause that leads to unilateral presentation, such as in the present case, remains unknown.

Although IIH is a relatively benign disease, it may be associated with visual loss from progressive retinal nerve fiber atrophy. Choroidal abnormalities, on the other hand, are rare but well-documented in the form of choroidal folds or choroidal neovascularization^(4,8-10)^. Our case is unique since it documents, for the first time, a PCV in IIH. On fundoscopy, PCV is characterized by reddish-orange subretinal nodules with serous retinal detachment and multiple hard exudates or large hemorrhage^(11)^. ICGA usually demonstrates, hyperfluorescent spots that originated from polypoidal dilatations of the choroidal circulation with or without a feeder and a draining vessel^(11)^. OCT reveals dome-shaped or thumb-like RPE elevation with or without internal polyps, the double-layer sign (a hyperreflective RPE separated from the Bruch’s membrane by a hyperreflective tissue), and dilated Haller’s layer vessels (pachyvessels) or pachychoroid^(11)^.

Although the pathogenesis of PCV remains uncertain, it is believed that pachyvessels at the Haller’s layer may attenuate the overlying choriocapillaris, impair superficial choroidal vessel flows, and generate a pro-angiogenic environment^(11)^. In papilledema, the anterior displacement of the optic nerve head and adjacent peripapillary tissues may compress or lead to ischemia^(12)^. It is possible that the chronic posterior globe flattening associated with intrinsic choroidal vessel abnormalities may have led to the development of the PCV in our patient.

Anti-vascular endothelial growth factor agents (anti-VEGF), with or without photodynamic therapy, is the standard treatment for PCV^(11)^. In our case, we opted for the anti-VEGF monotherapy combined with the standard treatment for IHH. The PCV became inactive with no recurrence in a 7-year follow-up period. Considering that the total regression of polyps is uncommon with anti-VEGF monotherapy, the inactivity of PCV in a long follow-up period suggests the important role of IIH treatment in PCV stabilization in the present case.

The intracranial meningioma could potentially be a confounding factor for the diagnosis of IIH. However, in our case, it neither caused a mass effect with brain edema nor interfered in the CSF dynamics or the venous drainage of the cerebral sinuses and therefore was not responsible for the increased ICP. Obesity is a common finding in patients with IIH, and weight loss can improve the disease prognosis, although the pathophysiology of its association is not well understood yet^(13)^. The current case is important as it draws attention to the possible association of unusual conditions including unilateral papilledema and the development of peripapillary PCV in a patient with IIH. Adequate knowledge of these possibilities and the exact role played by each of the involved aspects are of extreme importance for avoiding diagnostic confusion and inadequate treatment.
